# Supplementary material for: How the ‘plates’ of a health system can shift, change and adjust during economic recessions: A qualitative interview study of public and private health providers in Brazil’s São Paulo and Maranhão states
Source: PLoS One. 2020 Oct 26;15(10):e0241017. doi: 10.1371/journal.pone.0241017 (PMC7588110; doi:10.1371/journal.pone.0241017)
Supplement: S1 Annex — Questionnaire used for semi structured interviews (original version in Portuguese). (DOC) [file pone.0241017.s001.doc]

**S1 Annex: Interview guide (original version in Portuguese)**

# Roteiro de entrevista

Atualizado em: 13 de Dezembro de 18

# Introdução

Bom dia, somos uma equipa de pesquisa internacional da UFMA, USP e QMUL, e estamos a conduzir um estudo sobre os desafios trazidos pela situação económica do país para o setor saúde. Gostávamos que nos ajudasse a entender este tema contando-nos a sua opinião e experiência pessoal.

O estudo tem a autorização do Comité de Ética em Pesquisa de XXXX, e pedimos para gravar a entrevistas, garantindo confidencialidade da informação que o senhor/a nos querará comunicar, e a anonimidade. Em qualquer momento o senhor/a poderá pedir de desligar o gravador e pedir para ser retirado da entrevista.

## Qual é a sua idade, formação e há quanto tempo trabalha no setor?

## Pode explicar-nos quais são as suas funções de trabalho no setor saúde neste momento?

# Desafios económicos e do mercado do trabalho

## Nos últimos 10 anos a situação económica do país tem sofrido oscilações; você acha que mudou alguma coisa no seu trabalho como efeito direto ou indireto destas oscilações?

## Acha mudou alguma coisa na demanda de serviços da população?

### Sondar: na quantidade de doentes?

### Sondar: no perfil de utilizadores – mais pobre e desempregados? Classe média que perdeu planos?

## Você acha que mudou alguma coisa no seu trabalho como consequência da contração económica?

### Sondar: Aumentou a sua carga de trabalho?

### Sondar: Ao nível dos recursos no seu serviço (equipamentos, medicamentos, financiamento)?

# Medidas de governo na saúde

## Na sua opinião, quais terão sido as ações e decisões do governo que terão afetado mais o setor saúde nos últimos 10 anos?

## Quais terão sido as mudanças mais significativas no SUS nos últimos anos?

### Sondar: Diminuição de recursos federais e estaduais

### Sondar: Mudanças de prioridades como atenção primária, ou expansão da rede

### Sondar: Ações judiciárias e judialização da saúde

## Como se terão alteradas as Organizações de Saúde (OS)?

### Sondar: novos modelos de contratação e avaliação de desempenho

## Quais terão sido as mudanças mais significativas para os Planos de Saúde?

## E para os grandes hospitais privados?

### Sondar: Porquê em Maranhão não terão crescido mais?

## E para as Clínicas Populares?

### Sondar: porquê só agora é que estas clínicas terão começado a crescer e não antes?

## Terá havido mudanças importantes para outras instituições do setor saúde? Quais?

# Ajustes do setor

1. Acha que o setor se terá re-ajustado dalguma forma a estas mudanças?

### Sondar: o fluxo dos doentes mudou, eles entram no sistema de outra forma (UPAs, Clínicas Populares, ações judiciárias)

1. Come resultado direto ou indireto das mudanças alterações de condições que mencionou, o senhor/a acha que melhorou a capacidade de resposta do setor saúde?

Sondar: aumento/diminuição da cobertura e da qualidade

# Conclusão/ sugestões e agradecimentos

Muito obrigado pela informação prestada, vou desligar agora o gravador.

Será qua há mais algum elemento relevante que nos queria sugerir investigar sobre este tema? Com quem acha que deveríamos falar para lograrmos uma visão mais ampla deste tema?

Se estiver interessado/a nos resultados do estudo, podemos oferecer uma retro-informação; indique-nos para onde enviar dita informação.

Muito obrigado pela sua valiosa contribuição.
