# Supplementary material for: How the ‘plates’ of a health system can shift, change and adjust during economic recessions: A qualitative interview study of public and private health providers in Brazil’s São Paulo and Maranhão states
Source: PLoS One. 2020 Oct 26;15(10):e0241017. doi: 10.1371/journal.pone.0241017 (PMC7588110; doi:10.1371/journal.pone.0241017)
Supplement: S2 Annex — Questionnaire used for semi structured interviews (English version). (DOCX) [file pone.0241017.s002.docx]

**S2 Annex: Interview guide (translated in English)**

**Introduction**

Good morning, we are part of an international research team (USP, UFMA and QMU) that conducts a study on the impacts of the economic crisis on the Brazilian health system. We would like you to help us understand this topic, with your personal and professional opinion and experience.

(With ICF in hand)

The study is authorized by the Research Ethics Committee of our University. We ask for your authorization to record and guarantee the anonymity of your interview, which will be used only for the purposes of the study.

Perhaps you can tell us your age, training, career?

What are your current health roles and work?

# Labour market and new demands

1. In the last 10 years, the country's economic situation has fluctuated. Do you think that something has changed in your work, as a direct or indirect effect of these changes?

Probe: Has your job profile changed? workload? types of demands in the exercise of their professional activities?

Probe: in relation to resources involved in your work (services, equipment, medicines, financing, human resources)?

1. Do you think anything has changed in the population's demand for services and assistance? In the public sector? In the private sector?

Probe: Has there been a change in the types of demands and health problems? number of patients?

Probe: changing the profile of users (poorer? More unemployed? People who have lost health insurance?).

# Government health measures

1. In your opinion, what government actions and decisions have most affected the health sector in the past 10 years?
2. What were the most significant changes in SUS in recent years?

Probe: decrease of federal and state resources

Probe: changes in priorities (primary care? Network expansion? Policies and programs? In the role of the federal government, city halls and state governments?)

Probe: judicialization of health (lawsuits against SUS)

1. Were there changes in the participation and role of Health Organizations (OS)?

Probe: new forms of OS / public power relationship? new service management models? new ways of hiring staff? performance evaluation

1. Regarding the performance of health plans and insurance, have there been any changes?

Probe: in the relationship with providers (doctors, hospitals, laboratories)? payment methods for medical services? in “verticalization” (own service network)? Lawsuits against the plans?

1. What about private hospitals?

Probe: Have your relations with the SUS changed? with the plans? with doctors and professionals?

1. What do you think about the phenomenon of Popular Clinics?

Probe: are they expanding? why?

Probe: and the “discount cards” in consultations and exams

1. Do you identify changes in the performance of other sectors and institutions that work in health? Which are?

# Adjustments and adaptations of the health sector to changes

1. Do you think the health sector has already adapted in some way to these changes? In your opinion, what changes would be temporary, occasional, or which ones tend to keep in place?

Probe: Has the flow of users changed? do they enter the health system in other ways? Were there any changes (primary care, emergency care, specialties, Popular clinics, demands for lawsuits)?

Probe: Have labour relations changed? (links, forms of hiring, remuneration, workload)

1. As a direct or indirect result of the changes and alterations that you mentioned, do you think that the health sector's ability to respond has improved / worsened?

Probe: increase / decrease in coverage? quality? service functioning changes? in the performance of human resources? population satisfaction

# Conclusion and acknowledgements

To conclude, would you have suggestions on some other issue that we can investigate on this topic? Suggests a name / person / institution with whom we should speak to broaden our vision on this topic?

If you are interested, we are committed to giving you feedback on future results

of the study.

Thank you very much for the interview and for your valuable contribution. I will now turn off the recorder.

(Make sure that the informed consent form is signed. Give a copy to the interviewee).
