# Supplementary material for: How the ‘plates’ of a health system can shift, change and adjust during economic recessions: A qualitative interview study of public and private health providers in Brazil’s São Paulo and Maranhão states
Source: PLoS One. 2020 Oct 26;15(10):e0241017. doi: 10.1371/journal.pone.0241017 (PMC7588110; doi:10.1371/journal.pone.0241017)
Supplement: Attachment — Submitted filename: Response to editors and reviewers_011020.docx [file pone.0241017.s003.docx]

# 1. Editor’s further request

Please ensure that you refer to Table 2 and Table 3 in your text as, if accepted, production will need this reference to link the reader to the Table.

Response: This has been done in the paragraphs right before table 2 (pag.5) and table 3 (see pages 10-11).

# 2. Editor’s comments

1. Please ensure that your manuscript meets PLOS ONE's style requirements, including those for file naming.

Response: Headings and subheadings have been changed through the manuscript to meet the journal’s style requirements. All changes are highlighted in yellow in text.

2. Please address the following:

- Please include a copy of the interview guide used in your study in both the original language and English, as Supporting Information.

Response: Copies of the original language and translated interview guide have been uploaded as S1 and S2, respectively

- Please ensure you have thoroughly discussed all potential limitations of this study within the Discussion section, including the potential introduction of bias during data collection or sampling.

Response: We have now added a paragraph in the Discussion’s limitations section addressing the issue of bias and representativeness in our interview sample (Discussion, 2^nd^ par. pag.17.).

3. Please include captions for your Supporting Information files at the end of your manuscript, and update any in-text citations to match accordingly. Please see our Supporting Information guidelines for more information: [http://journals.plos.org/plosone/s/supporting-information](about:blank).

Response: Captions have now been introduced for our Supporting Information files S1 Annex and S2 Annex (pag.25).

# 3. Reviewer #1:

I found the article quite well developed and very well structured. Compatible with the initial purposes. The topic is very important and deserves, in my view, future development, especially due to the recent events related to the ongoing health crisis in Brazil. Perhaps a longer-term follow-up to a study on the actions of both SUS and the private sector, as well as their interactions.

Response: We thank reviewer 1 for this king comment. Please note this qualitative paper is part of a wider MRC project on the impact of the 2016-2018 economic recession in Brazil, and we are currently in the process of re-positioning its objectives to cover the impact of COVID-19.

# 4. Reviewer #2:

I believe that the method was appropriate for the study and for the question presented, although Table 2 "Strategies and quotes on the changing health insurance market in São Paulo and Maranhão” could be better measured and presented.

Response: We have now polished the translation and streamlined the topics in Table 2 (pag.12-14).

I consider one of the interesting points of the article the presentation of the different perceptions of the concept of "health sector crisis" among health care provider managers in São Paulo and Maranhão. It is interesting because such a perception may be strongly aligned with the respondents ' answers to the research questions, depending on their affiliation to the public or private sectors and also, how they understand the impacts of the economic recession on the health sector. In this sense, this point could be better explained for the profile of the interviewees. The profile of the interviewees was pointed out by the authors with a balance between workers of the public and private sectors, managers and professionals, capital and country cities, but the suggestion is that they explain more clearly the performance of the interviewees in the wide public and private health care systems. For example, do the doctors work in clinics, in basic health care services, in hospitals, etc.? I think that a clearer profile of the interviewees gives the reader a broader understanding of the perceptions found in the study.

Response: This is a valid point. However, rather than the professional profile of the respondents or their level of service in the health system, it was the public Vs private sector divide, and the SP Vs Maranhão distinction, that appeared to influence our respondents’ perceptions of crisis. We have now added this point in the Conflicting Perceptions of Crisis section (pag.8, first bullet point).

I suggest that the authors pay close attention to:
1) The Brazilian states represented in the study, São Paulo and Maranhão, are evidently diverse in their economic development process, and the coverage of access to health plans is characterized in this difference, as the authors cite. I suggest that they expand the characterization of the public network - SUS with more data from the two states, considering basic health care services, family health strategy, medium and high complexity, among others. This characterization, even if summarized, can broaden the understanding about the difference in the impacts of economic recession and health in São Paulo and Maranhão.

Response: We have now introduced a table in the Background section with an overview of key health and financing indicators for the two states and for the whole country (Tab. 1, pag. 4).

2) The concepts of systemic complexity and social systems (Niklas Luhmann and other authors) point out that there is a logic of singularities of functionality, construction and interrelation between them. In the case of the central argument of the article, which seeks to understand the changes and impacts of the crisis and recession on the public and private components and their derivations from the health care system, I think that supporting a reflection on the functional and social singularities and the relationship between them is important for the intended analysis. The complexity, in the systemic sense, of the public and private systems, when highlighted, strengthens the reflection on the results.

Response: We accept the argument about the relevance of Luhmann’s work for some of the arguments made in our paper. However, none of the authors has the sociology expertise necessary to apply and develop this kind of analysis. We have nonetheless inserted a reference in the Discussion about this body of work, noting that other disciplines may help understand better the effects of the crisis on the health system, particularly on the articulation and functions performed by its private and public and private components (Discussion, first paragraph, pag.18).

3) I consider it appropriate to consider the discussion about the covid-19 pandemic in the country and the possible impacts on health policies in Brazil, connecting it to the results of the research. However, I believe that an expansion of the analysis in this respect is necessary. The authors warn, in view of the results of the study, about the fact that some observed changes can last longer than the crisis itself, which is a very important issue to highlight. They also point out that it will be important to monitor the adjustments of the health care system to ensure that previous gains in access to care will not decrease. In this regard, do any signs after 5 months of the arrival of the pandemic in Brazil indicate losses, changes and expansion in the sectors studied?

Response: This paper is part of a wider study on the effects of the economic crisis on the health system in Brazil. We have now added a paragraph reflecting on some of our latest findings on the long-lasting effects of COVID-19 on the system (Discussion, last paragraph, pag. 18).

4) The Proposed Constitutional Amendment (PEC 95) on health financing, among other sectors, has shown harmful results for the SUS and the right to health, and highlighting those impacts in the text seems appropriate.

Response: We have now added a paragraph outlining features and consequences of PRC 95 (Introduction and Background, par.3, pag.3).

The article is very interesting and has significant results to discuss policies and the right to health in times of pandemic. The suggestions indicated in the review seek to strengthen the arguments and analyses presented by the authors.

Response: We thank the reviewers for their helpful and insightful comments.
